# How Knowledge Structure and Form Shape Scientific Divergent Thinking: Evidence from Semantic Network Analysis and the Scientific Divergent Application Task

**DOI:** 10.3390/jintelligence14070118

**Published:** 2026-06-25

**Authors:** Wenjin Zhang, Yoed N. Kenett, Mujie Ma, Peiduo Liu, Wenjing Yang

**Affiliations:** 1 Key Laboratory of Cognition and Personality, Southwest University (SWU), Ministry of Education, Chongqing 400715, China; wenjin@email.swu.edu.cn (W.Z.); swummj@email.swu.edu.cn (M.M.); 2 Faculty of Psychology, Southwest University (SWU), Chongqing 400715, China; 3 Faculty of Data and Decision Sciences, Technion-Israel Institute of Technology, Haifa 3200003, Israel; yoedk@technion.ac.il

**Keywords:** scientific creativity, scientific divergent thinking, semantic memory networks, knowledge structure, knowledge forms

## Abstract

Scientific creativity depends not only on what learners know but also on how knowledge is organized, yet evidence in scientific domains remains limited. Across two experiments, we examined whether knowledge structures predict scientific divergent thinking and whether knowledge form shapes creativity through its effects on these structures. Undergraduate students completed the Scientific Divergent Application Task (SDAT), which requires generating novel applications based on learned scientific principles. In Pilot Experiment (*n* = 39), semantic memory networks constructed from participants’ knowledge representations revealed that network connectivity and efficiency were positively associated with better SDAT performance, whereas recall accuracy was unrelated. In Experiment 2 (*n* = 126), holding informational content constant, knowledge forms significantly influenced performance: both associative and schematic knowledge promoted scientific divergent thinking more effectively than case-based knowledge, whereas schematic knowledge further demonstrated advantages in originality and knowledge network connectivity and efficiency. These findings suggest that knowledge organization, rather than retention alone, underlies individual differences in scientific creativity, and that schematic presentation may support creative application by fostering more efficient knowledge structures.

## 1. Introduction

Scientific creativity is defined as the ability to generate ideas that are both novel and appropriate in scientific contexts. It is increasingly viewed as a core outcome of STEM education and a prerequisite for addressing complex, uncertain real-world problems (Prahani et al., 2024; Hu & Adey, 2002; Eroğlu & Bektaş, 2022). As digital technologies and artificial intelligence progressively externalize knowledge acquisition, storage, and retrieval, educational goals can no longer be centered solely on mastery and recall of factual content (Clark, 2025). Instead, a central challenge for STEM learning is to enable learners to reorganize what they know and transfer it to generate innovative applications within scientifically constrained problem spaces.

Knowledge has long been considered indispensable for creativity, yet its role is paradoxical (Kenett, 2025; Yang et al., 2022). On the one hand, domain knowledge provides the cognitive substrate for understanding scientific problems and generating plausible solutions (Abraham & Bubic, 2015; Resch & Kock, 2021). On the other hand, existing knowledge may also induce mental set, narrowing the search space and biasing individuals toward conventional frameworks (Nagle & Teodoridis, 2020; Wiley, 1998). This contradictory phenomenon suggests that the impact of knowledge on creativity may depend less on how much knowledge a person possesses than on how knowledge is represented and organized in memory (Benedek et al., 2023; Kenett, 2025). The associative theory of creativity posits that highly creative individuals have semantic memory structures that facilitate access to remote concepts (Kenett, 2025; Mednick, 1962). For a long time, methodological constraints limited researchers’ ability to quantify knowledge representations with objective metrics (Kumar, 2021), leading many studies to rely on indirect indicators (e.g., educational background, test scores, expert–novice distinctions) or subjective assessments such as concept maps (X. Liu et al., 2025; Jackson et al., 2023; Teng et al., 2022; Kinchin et al., 2021; Karwowski et al., 2020). Recent advances in network science have made it feasible to model knowledge representations as networks in which concepts are nodes and their relations are edges, and to characterize structural properties using metrics such as clustering and efficiency (Castro & Siew, 2020; Haim & Stella, 2026; Hills, 2025). Yet, most evidence linking semantic memory network structure to creativity comes from domain-general creative thinking, and scientific creativity remains comparatively understudied (Beaty et al., 2025, 2026). Thus, a key gap is whether objectively quantified knowledge structure is reliably associated with creative performance in a knowledge-rich scientific context, where novelty must remain scientifically plausible.

From an educational perspective, an equally important question concerns the instruction: If knowledge structure matters, how can educators support learners in develop structures that better enable scientific creative ideation? One prominent instructional lever is the form in which knowledge is presented. In practice, scientific content can be communicated as schematic, principle-based explanations; as associative materials that link new ideas to familiar concepts; or as case-based narratives that embed principles in concrete situations. Prior work indicates that knowledge form can shape learning and subsequent application, and some studies have examined knowledge form effects on creativity (Luchini et al., 2024; Siew & Guru, 2023). However, evidence largely focuses on domain-general divergent thinking or creative problem solving, and often conflates form with differences in informational content (Afkar et al., 2024; W. Liu et al., 2020). Therefore, a second gap is whether knowledge form, when content is held constant, systematically changes scientific divergent thinking, and whether these effects can be explained by the knowledge structures learner’s construct.

To address these gaps, the present study uses the Scientific Divergent Application Task (SDAT) to simulate a learning-to-innovation sequence in a knowledge-rich scientific domain (Zhou et al., 2026). The SDAT requires participants to learn the basic principles of recently developed scientific inventions/materials and then generate diverse, original applications that remain consistent with the learned constraints. This design allows us to examine creativity as knowledge application rather than ideation divorced from content. Across two experiments, we ask: (1) How are individual knowledge structures associated with scientific divergent thinking? (2) Do different knowledge forms (schematic vs. associative vs. case-based), with the same core information, shape scientific creativity by influencing knowledge structures?

Pilot Experiment focused on individual differences, examining whether objectively quantified knowledge-structure properties using semantic memory network methods and graph-theoretic metrics (e.g., clustering coefficient, global efficiency, local efficiency and average shortest path length) were associated with performance on the SDAT. Drawing on network accounts of semantic memory, we hypothesized that more integrated and efficient knowledge structures would be associated with better scientific divergent thinking, reflected in higher fluency, flexibility, and originality (Kenett, 2025), beyond simple knowledge retention). Experiment 2 adopted an instructional manipulation approach, holding core content constant while varying knowledge form (schematic, associative, or case-based). From an instructional perspective, schematic representations emphasize abstract causal structure and constraints, which should promote the construction of more interconnected and efficient knowledge networks than associative or case-based presentations that may anchor knowledge in local links or surface contexts (Z. Zhang et al., 2025; Yuan et al., 2023; Hao & Wu, 2010). Therefore, we hypothesized that schematic knowledge presentation would foster more integrated and efficient knowledge structures than associative or case-based formats and would be associated with higher SDAT performance.

## 2. Pilot Experiment

Pilot Experiment aims to quantify individual knowledge structures using semantic memory network construction methods, preliminarily exploring the relationship between knowledge structure and scientific creativity. Based on prior research (Kenett, 2025), we hypothesize that individuals who develop more efficient knowledge structures following learning will demonstrate higher levels of scientific creativity. In particular, higher clustering coefficients, greater efficiency, and shorter average shortest path lengths are expected to predict better performance.

### 2.1. Participants

Thirty-nine undergraduate students (10 males, 29 females) from non-psychology majors were recruited online from a university. Participants were 19–25 years old (*M* = 20.87 years, *SD* = 1.30 years). All participants were right-handed, had normal or corrected-to-normal vision, reported no history of neurological or psychiatric disorders, and had not participated in similar experiments. Participants received monetary compensation upon completion. The study protocol was approved by the institutional review board of the affiliated institution, and all participants provided informed consent.

### 2.2. Material

Scientific Divergent Application Task (SDAT). The SDAT is designed to simulate the real-world process of scientific invention, in which existing technologies are transferred to novel application domains to generate innovative solutions. For example, imaging sensor technology developed for the Hubble Space Telescope program contributed to advances in consumer digital photography, and additive manufacturing (3D printing), initially developed for military aerospace applications, later yielded transformative breakthroughs across multiple industries.

In the SDAT, participants are presented with descriptions of advanced materials or technologies with specific properties and are asked to generate potential applications based on these properties (i.e., “Given these characteristics, what other applications could this material/technology be used for?”). Materials were selected from a large corpus of recent inventions collected from the internet, newspapers, and academic journals. Selection criteria emphasized novelty, fundamental operating principles, and real or potential application domains, prioritizing materials, technologies, and devices with broad transfer potential. The learning materials used in the SDAT are provided in Supplementary File S1.

The SDAT is intended for use in STEM educational contexts to assess general scientific creative thinking while minimizing dependence on specialized disciplinary knowledge (e.g., chemistry or engineering). The task consists of two main phases (Yang et al., 2022): during the learning phase, participants study the characteristics and functions of a novel material or invention; during the creative thinking phase, they generate novel and diverse applications based on the learned information.

An example stimulus used in this experiment described A alkene, a nearly transparent novel material that is reportedly among the lightest and hardest substances known, with excellent electrical and thermal conductivity. Example applications included components for supercomputers to enhance electron mobility; sensor-surface coatings combining lightness, thinness, and transparency; smartphone screens balancing strength with thermal conductivity; and bulletproof vests that reduce bulk while increasing toughness and strength.

### 2.3. Procedure

**Figure 1:**
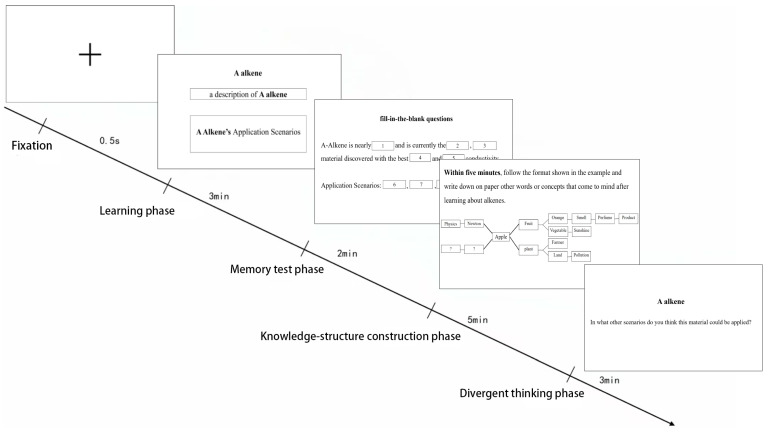
Flowchart of the Scientific Divergent Application Task in Pilot Experiment.

The SDAT was programmed and administered in E-Prime. Participants completed the experiment individually in a quiet testing room. All stimuli were presented on a computer monitor, and responses were recorded automatically by the system. To examine links among learning retention, knowledge-structure organization, and creative output, we included a memory test and a knowledge structure construction phase prior to the divergent thinking phase. The experiment consisted of four phases (Figure 1):

Learning phase. A fixation cross (“+”) appeared for 0.5 s to signal the start of the trial, followed by a description of the novel material/technology. Participants studied, understood, and memorized the information within 120 s.

Immediate recall test phase. Participants completed a fill-in-the-blank test assessing recall of key information from the learning phase. They had 2 min to complete the items.

Knowledge-structure construction phase. Participants constructed a concept map to externalize their knowledge structure. They were instructed to write down at least 15 pieces of knowledge associated with the newly learned material on paper within 5 min and to depict the relationships among these concepts (i.e., how they are interconnected). Specifically, participants created a mind map-diagram in which each concept was represented as a node, and relationships were depicted by drawing links between concepts. There were no constraints on the hierarchical structure, allowing participants to freely organize concepts at multiple levels. Participants were not required to label the links with specific relational descriptions; the presence of a link was taken to indicate a perceived connection between concepts.

Divergent thinking phase. Participants generated applications for the newly learned material beyond those provided in the description. They had 3 min to respond.

To further evaluate the convergent validity of SDAT performance, participants also completed the Alternative Use Task (AUT; Guilford, 1967), a classic measure of divergent thinking. Participants were asked to generate as many novel uses as possible for two common everyday objects (an aluminum can and an empty paper box), with 3 min allocated for each object. The order of task completion (AUT and SDAT) was counterbalanced across participants, such that half of the participants completed the AUT first and the other half completed the SDAT first.

### 2.4. Data Analysis

#### 2.4.1. Scoring of SDAT and AUT Responses

After excluding invalid responses (meaningless or incomprehensible answers), three raters scored the responses across four dimensions: (1) Overall: A composite score of 1–10 representing the participant’s overall creativity, evaluated by integrating scores across all dimensions (Silvia et al., 2009). (2) Flexibility, measured by the number of distinct categories into which the participant’s responses could be grouped, with each category scoring 1 point; the sum of all categories constituted the participant’s flexibility score. (3) Fluency, measured by the number of valid responses generated by the participant, with each valid response scoring 1 point; the sum of all valid responses constituted the participant’s fluency score. (4) Originality, which involves rating the uniqueness of each individual response on a scale of 1 to 10, where 1 indicates no originality and 10 indicates high originality. The sum of all response scores constitutes the participant’s originality score (Forthmann et al., 2020; Saretzki et al., 2025). Rater agreement was high among the three evaluators, with intraclass correlation coefficients (ICC) ranging from 0.70 to 0.89 for the SDAT and from 0.76 to 0.98 for the AUT.

#### 2.4.2. Construction of Knowledge-Structure Networks and Graph-Theoretic Metrics

The calculation of knowledge structure indicators involves two stages: (a) knowledge structure construction and (b) graph metric computation for the knowledge structure. During the knowledge structure construction phase, the semantic distance between pairs of words within the participant’s knowledge structure are computed. This study employs the Word2Vec toolkit (https://code.google.com/archive/p/word2vec/, accessed on 11 May 2025) integrated into Python 3.10 to compute semantic distances between words. This toolkit utilizes Chinese corpora from Wikipedia and Baidu Baike, comprising approximately one billion word tokens. First, all non-Chinese characters were removed, and traditional Chinese characters were converted to simplified Chinese. The corpus was then segmented using the Python-based Jieba Chinese parser, yielding a final vocabulary of 1.53 million words. Finally, word2vec was employed to train word vectors with a dimension of 300. The cosine value of the angle between the word vectors of two words represents their semantic similarity. Subtracting this semantic similarity value from 1 yields a metric for their semantic distance. A knowledge structure network was then constructed based on semantic distances between words. For each subject, all connections among 15 key words were retained, forming a 15 × 15 weighted undirected network (WUN; Benedek et al., 2017). Based on the semantic distance computation described above, participants’ concept maps were transformed into weighted semantic memory networks, in which edge weights reflected the semantic distance between word pairs derived from the Word2Vec model.

Graph theory metrics for knowledge structure were analyzed using the MATLAB-based Brain Connectivity Toolbox, release 2019-03-03 (BCT; https://sites.google.com/site/bctnet/, accessed on 11 May 2025), in MATLAB R2020a, for network graph analysis (Rubinov & Sporns, 2010). Four common graph theory metrics—clustering coefficient, local efficiency, global efficiency, and average shortest path length—were computed for each knowledge structure network (Hills, 2025; Wang et al., 2015; Rubinov & Sporns, 2010). The clustering coefficient (CC) measures the probability that neighbors of a node i are neighbors of each other (where neighbors are defined as nodes connected by a direct link). It represents the ratio of the actual number of connections between a node’s neighbors to the number of possible connections, quantifying the closeness of adjacency among network nodes. Local efficiency (Eloc) calculates the shortest distance between any two nodes in the subgraph formed by the neighbors of node i. A high local efficiency indicates faster and more efficient information propagation between a node and its neighbors. A network’s local information transmission capability can be represented by local efficiency and clustering coefficient. Global Efficiency (GE) refers to the average reciprocal of the shortest path length in a network (Latora & Marchiori, 2001). It is one of the metrics used to measure the efficiency of information transmission within a network. A high global efficiency indicates faster and more efficient information propagation within the network. Average Length of the Shortest Path (ASPL) represents the average number of steps required for information to propagate from one node to another within a network. A shorter ASPL indicates faster information dissemination and tighter connections between nodes.

### 2.5. Results

#### 2.5.1. Convergent Validity: SDAT Performance Was Positively Correlated with AUT Performance

First, we examined the SDAT convergent validity in the present study using the classic AUT as a criterion measure. As shown in Table 1, SDAT scores were significantly and positively correlated with AUT scores across all corresponding dimensions (i.e., overall creativity, fluency, flexibility, and originality), supporting the convergent validity of the SDAT in this sample.

#### 2.5.2. SDAT Performance Was Not Significantly Associated with Recall Accuracy

Next, participants’ learning retention was indexed by accuracy on the fill-in-the-blank items administered immediately after the learning phase. Overall recall was high (*M* = 87.08%, *SD* = 0.15), indicating that participants generally remembered the newly learned information. To test whether retention was associated with scientific divergent thinking, we correlated recall accuracy with SDAT performance. Recall accuracy was not significantly correlated with SDAT overall, *r* = −0.010, *p* = .950; fluency, *r* = 0.001, *p* = .995; flexibility, *r* = −0.049, *p* = .769; or originality, *r* = −0.020, *p* = .906; suggesting that individual differences in SDAT performance were not explained by differences in immediate factual recall. This pattern suggests that the observed associations with creativity are more likely attributable to how knowledge is organized rather than how accurately it is recalled.

#### 2.5.3. Associations Between SDAT Performance and Quantified Knowledge-Structure Indices

To examine the relationship between knowledge structure and scientific divergent thinking, we quantified participants’ knowledge representations using semantic memory network methods (Kenett, 2025). We then conducted correlation analyses between graph-theoretic metrics derived from each participant’s knowledge-structure network and SDAT performance. The clustering coefficient, global efficiency, and local efficiency were positively correlated with SDAT overall, fluency, and flexibility (Table 2). In contrast, average shortest path length was negatively correlated with SDAT overall, fluency, and flexibility. Together, these results suggest that more efficiently organized knowledge structures, characterized by higher local clustering and greater global and local efficiency, are associated with better scientific divergent thinking performance. These findings are consistent with the hypothesis that network efficiency and local clustering capture functional aspects of knowledge organization that support flexible idea generation (He et al., 2025; Kenett, 2025; Kenett & Beaty, 2023).

### 2.6. Discussion

Pilot Experiment examined the association between individual knowledge structures and scientific creativity as assessed by the SDAT. We first re-established the SDAT’s effectiveness by benchmarking it against the AUT, a widely used measure of general divergent thinking. We then compared two ways of characterizing newly acquired knowledge: traditional recall performance (accuracy on fill-in-the-blank items) and semantic memory network indices derived from participants’ concept-based knowledge representations. The results revealed a clear divergence in predictive utility. Immediate recall test accuracy were high overall but did not account for individual differences in scientific creative performance, suggesting that merely remembering the learned information within a short period of time is insufficient to explain why some individuals generate more creative applications than others.

In contrast, the organization of knowledge, captured by semantic memory network properties, was reliably associated with SDAT performance. Participants whose knowledge networks exhibited higher clustering coefficients and shorter average shortest path lengths showed better creative performance following learning. Moreover, highly creative individuals’ networks were characterized by greater global and local efficiency, indicating a structure that supports both rapid integration across concepts and efficient local information transfer. This pattern aligns with prior evidence from general creativity research, showing that more efficient semantic memory networks are linked to superior creative performance (Kenett, 2025).

These findings underscore the importance of knowledge structure within semantic memory as a cognitive foundation for scientific creativity. In educational practice, instructors often attempt to enhance creativity by changing the external form of knowledge presentation (e.g., examples, visualizations, or instructional sequencing). However, an open question is whether, and to what extent, creativity gains in such contexts depend on the learner’s internal organization of knowledge (Luchini et al., 2024; Siew & Guru, 2023). Future work should therefore examine whether instructional manipulations that reshape learners’ knowledge structures lead to measurable improvements in scientific creativity, and whether knowledge-structure indices continue to predict creative performance when teaching interventions explicitly target knowledge organization.

## 3. Experiment 2

The results of Pilot Experiment suggest that variation in scientific creative performance is not explained by differences in recall accuracy, but is associated with differences in how information is organized within semantic representations. From an educational perspective, this implies that teaching strategies may enhance scientific creativity by altering the form of knowledge presentation to promote more efficient internal knowledge structures (Luchini et al., 2024; Siew & Guru, 2023). In Experiment 2, we compared three prevalent knowledge forms—schematic, associative, and case-based knowledge (Hao & Wu, 2010; Hunter et al., 2008)—and investigated their effects on scientific divergent thinking. We further tested whether knowledge-structure properties derived from semantic network analyses account for individual differences in creative outcomes under different instructional forms.

### 3.1. Participants

Experiment 2 employed a between-subjects, three-condition design (schematic vs. associative vs. case-based knowledge). An a priori power analysis was conducted using G*Power 3.1.9.2 for a one-way ANOVA. Assuming an effect size of *f* = 0.30, *α* = 0.05, and power = 0.80, the required sample size was estimated to be 111 participants. The final sample size of 126 therefore provided sufficient statistical power. One hundred and twenty-six undergraduate students from non-psychology majors were recruited (21 males, 105 females), aged 18–26 years (*M* = 20.88 years, *SD* = 1.49 years). All participants were right-handed, had normal or corrected-to-normal vision, reported no history of psychiatric or neurological disorders, and had not previously participated in similar experiments. Participants provided written informed consent and received compensation. The study protocol was approved by the institutional review board.

### 3.2. Materials and Manipulation of Knowledge Form

The SDAT materials were drawn from the Experimental Problem Materials Database for Scientific Invention and Creation (Yang et al., 2022). Following established definitions, schematic knowledge emphasized abstract principles, associative knowledge emphasized probabilistic/experiential linkages among concepts, and case-based knowledge emphasized an event-like mental model with contextual details (Hao & Wu, 2010; Hunter et al., 2008). We revised the original materials into these three forms while keeping the core informational content constant. Three trained raters evaluated the rewritten versions to ensure fidelity to the intended knowledge form and equivalence in core content. Below is an example of an explanation of A alkene in three different forms of knowledge.

Schematic Knowledge: A alkene is nearly transparent and stands as the lightest, hardest, and most conductive material discovered to date.

Associative Knowledge: A alkene is a novel material with multiple properties and potential applications across various fields: excellent conductivity—enhancing circuitry; lightweight and thin—enabling stretchable films; transparency—replacing glass and acrylic; high hardness—rivaling steel.

Case-based Knowledge: A alkene is a novel material with potential applications across diverse fields, such as: serving as wire cores to enhance electrical conductivity; improving performance in existing supercomputer components with low electron mobility; and forming lightweight, heat-dissipating, damage-resistant films when melted, suitable as coatings for next-generation sensors. When compressed, A alkene achieves high density while remaining crystalline and lightweight. Its excellent conductivity also grants high sensitivity, making it suitable for smartphone screens. As a fiber additive in synthetic materials, it enhances both toughness and strength, potentially replacing steel plates in bulletproof vests.

### 3.3. Procedure

**Figure 2:**
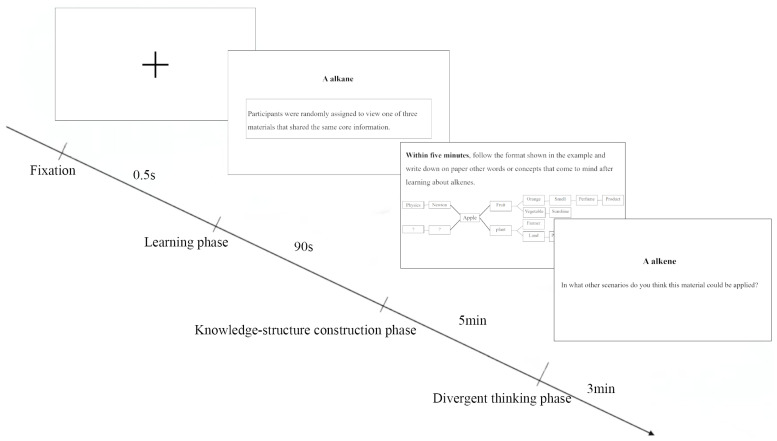
Flowchart of the Scientific Divergent Application Task in Experiment 2.

Participants were randomly assigned to one of the three knowledge-form conditions (schematic, associative, or case-based). They were tested individually on a computer, and responses were recorded. Based on Pilot Experiment performance and participant feedback, the learning phase was shortened to 90 s in Experiment 2 (Figure 2).

### 3.4. Data Analysis

#### 3.4.1. Measures and Scoring of Scientific Divergent Thinking

Responses were scored on four SDAT dimensions: overall, fluency, flexibility, and originality. To reduce collinearity between originality and fluency, Experiment 2 operationalized originality as the mean originality per response, following prior recommendations, rather than as a summed originality score (Forthmann et al., 2020). Other scoring procedures followed Pilot Experiment. Inter-rater reliability was high across dimensions (ICC = 0.80–0.97).

#### 3.4.2. Knowledge Structure Quantification

Knowledge structure indices were computed in two stages: constructing an individual semantic memory network from participants’ responses and then calculating graph metrics. Semantic distances between words were computed using a Word2Vec toolkit (integrated in Python) trained on large Chinese corpora (Wikipedia and Baidu Baike). Semantic similarity was estimated as cosine similarity, and semantic distance was defined as 1 − cosine similarity. For each participant, a weighted undirected network was constructed with 15 key words, retaining all pairwise connections, resulting in a 15 × 15 weighted undirected network.

Graph-theoretic metrics were computed using the MATLAB-based Brain Connectivity Toolbox (Rubinov & Sporns, 2010). Four commonly used metrics were calculated: clustering coefficient (CC), local efficiency (Eloc), global efficiency (GE), and average shortest path length (ASPL).

### 3.5. Results

**Figure 3:**
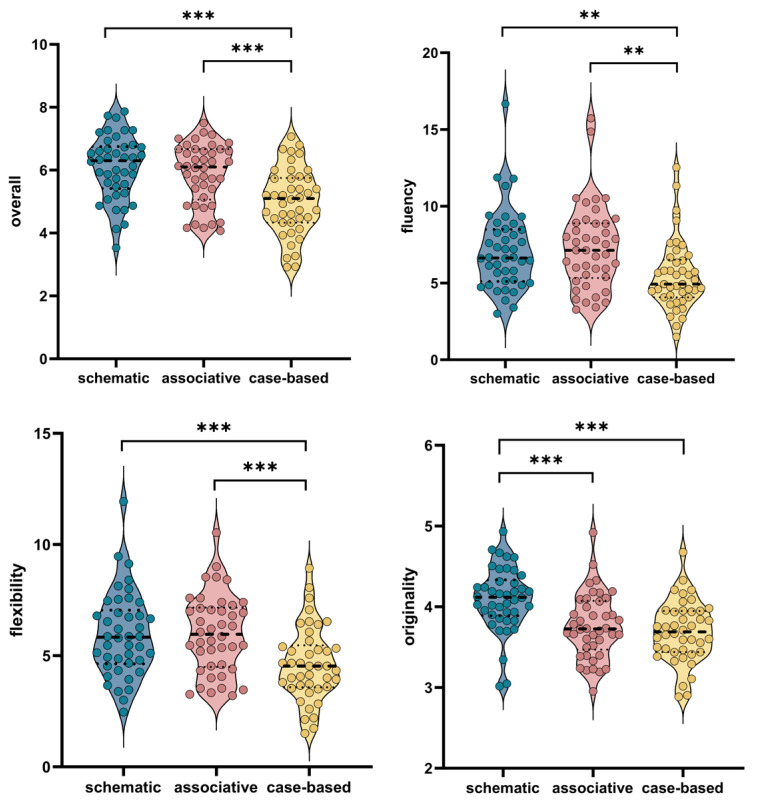
Scores on the SDAT across three forms of knowledge. SDAT stands for the Scientific Divergent Application Task. Violin plots represent the distribution of the data, with embedded boxplots indicating the median and interquartile range. Individual data points are overlaid. **—*p* < .01, ***—*p* < .001.

**Figure 4:**
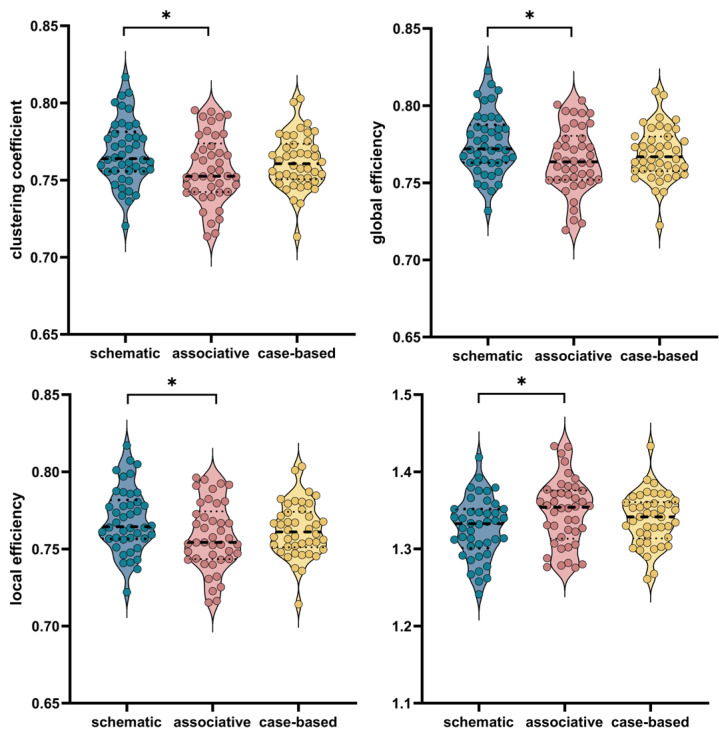
Scores on the SDAT across three forms of knowledge. SDAT stands for the Scientific Divergent Application Task. Violin plots represent the distribution of the data, with embedded boxplots indicating the median and interquartile range. Individual data points are overlaid. *—*p* < .05.

Preliminary analyses were conducted to examine whether the three groups differed in demographic characteristics. The results indicated no significant differences in gender distribution across groups, χ^2^(2) = 1.371, *p* = .504, or in age, *F*(2, 123) = 0.668, *p* = .515, suggesting that the groups were comparable prior to the experimental manipulation. We then tested the effect of knowledge form on SDAT outcomes using one-way ANOVAs (overall, fluency, flexibility, originality), followed by multiple comparisons, as shown in Figure 3. Next, we tested whether knowledge form altered knowledge-structure metrics (CC, GE, Eloc, ASPL) using one-way ANOVAs and follow-up comparisons (Figure 4). Finally, we examined the association between knowledge-structure metrics and SDAT outcomes to provide mechanistic evidence (Table 3 and Table 4).

#### 3.5.1. Effects of Knowledge Form on SDAT Outcomes

Given our theory-driven ordering (schematic/associative knowledge > case-based knowledge), we conducted a series of one-way ANOVAs on SDAT performance across the schematic, associative, and case-based conditions. Results indicated significant between-group differences for SDAT overall, *F*(2, 123) = 13.46, *p* < .001, η^2^ = 0.18; fluency, *F*(2, 123) = 6.51, *p* = .002, η^2^ = 0.10; flexibility, *F*(2, 123) = 7.98, *p* < .001, η^2^ = 0.11; and originality, *F*(2, 123) = 12.66, *p* < .001, η^2^ = 0.17.

Subsequent multiple comparisons (Figure 3) showed that the schematic condition yielded higher originality (*M* = 4.09, *SD* = 0.40, 95% CI = [3.97, 4.22]) than the associative condition (*M* = 3.76, *SD* =0.40, 95% CI = [3.64, 3.89]) and the case-based condition (*M* = 3.67, *SD* = 0.38, 95% CI = [3.57, 3.81]), *p* < .001. In addition, the schematic condition also outperformed the case-based condition on overall (*M* = 6.09, *SD* = 1.02, 95% CI = [5.78, 6.41], *p* < .001), fluency (*M* = 7.10, *SD* = 2.62, 95% CI = [6.28, 7.92], *p* = .004), and flexibility (*M* = 5.94, *SD* = 1.91, 95% CI = [5.35, 6.53], *p* < .001). The associative condition similarly outperformed the case-based condition on overall, fluency, and flexibility.

Overall, both schematic and associative formats supported higher SDAT performance than the case-based format on most dimensions, whereas schematic knowledge showed an additional advantage for originality.

#### 3.5.2. Effects of Knowledge Form on Knowledge-Structure Metrics

We next tested whether different knowledge formats produced systematically different knowledge structures. Although omnibus tests for the graph-theoretic indices did not reach conventional significance levels (clustering coefficient *F*(2, 123) = 3.06, *p* = .050, η^2^ = 0.047; global efficiency *F*(2, 123) = 3.00, *p* = .055, η^2^ = 0.046; local efficiency *F*(2, 123) = 3.01, *p* = .053, η^2^ = 0.047; average shortest path length *F*(2, 123) = 2.97, *p* =.055, η^2^ = 0.046), the theory-driven schematic-associative contrast showed a consistent directional pattern across all indices: schematic knowledge yielded higher clustering coefficient(*M* = 0.77, *SD* = 0.02, 95% CI = [0.76, 0.77]), global efficiency(*M* = 0.78, *SD* = 0.02, 95% CI = [0.77, 0.78]), and local efficiency (*M* = 0.77, *SD* = 0.02, 95% CI = [0.76, 0.78]), as well as shorter average shortest path length (*M* = 1.33, *SD* = 0.04, 95% CI = [1.31, 1.34]) than associative knowledge (*p* = .015–0.017; Figure 4). In terms of magnitude, schematic–associative differences were consistently medium across all graph-theoretic indices (Cohen’s *d* = 0.50–0.51; negative for shortest path length), indicating that schematic knowledge produced a more integrated and transmission-efficient representational structure. No significant differences were observed between the case-based knowledge condition and the other two knowledge forms in any of the graph-theoretic indices (all *p’*s > .05).

#### 3.5.3. Mechanistic Triangulation Linking Knowledge Structure to SDAT Performance

Finally, we evaluated whether the overall pattern of findings is consistent with a knowledge-organization account of scientific divergent thinking. Correlation analyses (Table 3) indicated that clustering coefficient, global efficiency, and local efficiency were significantly and positively correlated with SDAT overall, fluency, flexibility, and originality, whereas average shortest path length showed significant negative correlations with all SDAT dimensions. This replicated the results of Pilot Experiment in a larger sample.

To further examine whether knowledge structure statistically accounted for SDAT variance beyond knowledge form, we conducted hierarchical regression analyses. Knowledge form (dummy-coded) was entered in the first step, followed by knowledge-structure index in the second step. Because graph-theoretic indices of knowledge structure were moderately to highly intercorrelated, we constructed a composite knowledge-structure score by standardizing clustering coefficient, global efficiency, local efficiency and reverse-scoring average shortest path length before averaging them into a single knowledge structure index. Hierarchical regressions showed that adding the composite knowledge-structure index significantly improved model fit across SDAT dimensions (Δ*R*^2^ = 0.038–0.044, *p* = .010–.017). The knowledge structure index positively predicted SDAT performance (β = 0.199–0.215), while the coefficients of knowledge form were attenuated relative to baseline models. This pattern suggests that representational organization accounted for a meaningful portion of the performance differences associated with knowledge form.

To examine the robustness of the composite knowledge structure index, we conducted a principal component analysis (PCA) on the graph-theoretic indicators. The PCA yielded a single component accounting for 99.75% of the variance, with all indicators loading extremely strongly on this component (loadings = 0.998–1.000). Analyses using the PCA-derived scores produced identical results to those obtained using the composite index.

### 3.6. Discussion

Experiment 2 extends Pilot Experiment by moving from correlational evidence based on variability in participants’ knowledge structures to an instructional manipulation, providing a stronger basis for interpreting how learning materials may foster scientific divergent thinking. First, Experiment 2 replicated the structural pattern observed in Pilot Experiment: knowledge networks characterized by higher clustering coefficient, higher global and local efficiency, and shorter average shortest path length were associated with better SDAT performance across overall score, fluency, flexibility, and originality. This replication in a larger sample strengthens the reliability of the semantic-network approach for capturing knowledge-organization correlates of scientific creativity.

Second, manipulating knowledge form produced clear differences in scientific divergent thinking. When core content was held constant, schematic knowledge yielded the best SDAT performance, particularly in originality, and both schematic and associative formats outperformed the case-based format on most SDAT dimensions. This pattern differs from prior findings in domain-general creativity contexts where schematic and associative knowledge often show comparable effects (Hunter et al., 2008), suggesting that the benefits of specific knowledge forms may be domain-sensitive and particularly pronounced when creativity depends on principle-based constraints and scientific plausibility (Luchini et al., 2024; Siew & Guru, 2023).

Third, although a single mediation test did not provide definitive evidence for an indirect effect, the results jointly provide convergent, mechanism-consistent support for a knowledge-organization account. The schematic format not only improved SDAT outcomes but also tended to produce more clustered and efficient knowledge structures (higher CC/GE/Eloc and shorter ASPL), and these structural properties were robustly linked to SDAT performance. Taken together, Experiment 2 supports the interpretation that instructional form may facilitate scientific idea generation partly by shaping how newly learned scientific principles are organized and traversed during divergent application.

Taken together, Experiment 2 provides convergent mechanistic support based on three aligned findings. First, knowledge form influenced SDAT outcomes. Second, knowledge form also shaped knowledge structure properties, and more clustered and efficient knowledge structures (higher CC/GE/Eloc and shorter ASPL) were associated with better scientific divergent thinking performance. This triangulated pattern is consistent with the interpretation that representational form supports scientific divergent thinking partly by shaping the organization and efficiency of learners’ knowledge representations.

## 4. General Discussion

This study examined the creativity trajectory from learning to application in scientific contexts by using the Scientific Divergent Application Task (SDAT). The SDAT decomposes scientific creativity into a learning phase (knowledge acquisition), a representational phase (knowledge-structure construction), and an ideation phase (divergent application). Across two experiments, we asked (a) whether objectively quantified knowledge structures relate to scientific divergent thinking and (b) whether knowledge form, as a manipulable instructional feature, shapes scientific creativity and learners’ knowledge structures during learning.

### 4.1. Knowledge Structure and Scientific Divergent Thinking

A central contribution of the present work is to operationalize “knowledge structure” in a measurable way and demonstrate its relevance for scientific creativity. Building on computational cognitive network approaches (Haim & Stella, 2026; Hills, 2025), we treated concepts as nodes and semantic distances as edges, and quantified individual knowledge structures using graph-theoretic metrics. Across both studies, more clustered and efficient structures (higher CC/GE/Eloc) and shorter paths (lower ASPL) were systematically associated with stronger SDAT performance. Importantly, the structural account is consistent with an information-access view of creative ideation: knowledge networks with shorter paths and higher transmission efficiency may reduce the cognitive “distance” between concept clusters, facilitating flexible search and recombination during idea generation (He et al., 2025).

Our findings extend prior semantic memory network creativity research in two ways. First, existing work has focused primarily on domain-general divergent thinking; in contrast, the SDAT requires generating novel applications that remain scientifically plausible given learned constraints. Thus, the present study provides initial domain-specific evidence that semantic memory network organization is predictive not only of general creative association but also of scientifically constrained divergent application. Second, by embedding creativity within a learning-to-application sequence, we complement the standard approach of minimizing domain knowledge in divergent thinking tasks and instead treat knowledge organization as part of the creative process itself.

These results align with emerging evidence that semantic memory network structure is linked to creative cognition and real-world creative activities (Y. Zhang et al., 2025). Together, this growing literature suggests that semantic memory networks provide a tractable representational substrate for understanding why some individuals can more effectively navigate conceptual spaces to generate novel and useful ideas. The present study supports this account in the context of scientific creativity and highlights knowledge structure as a promising explanatory construct for STEM-related creative learning outcomes.

### 4.2. Knowledge Form and Scientific Divergent Thinking

A second major contribution is to connect knowledge-structure theory to an instructional lever: knowledge form. In real learning environments, the same scientific content can be communicated as schematic, principle-based explanations; associative linkages to familiar concepts; or case-based narratives with contextual details. Prior research has examined knowledge-form effects on creativity largely in domain-general and artistic creativity settings (Althuizen & Wierenga, 2014; Cheng et al., 2010; Choi & Kim, 2017; W. Liu et al., 2020), leaving scientific divergent thinking comparatively understudied. By rewriting the same scientific materials into schematic, associative, and case-based forms while preserving core meaning, we provide a more direct test of how representational form shapes scientific creative performance.

The results indicate a dimension-specific pattern in scientific divergent thinking, with both schematic and associative knowledge outperforming case-based knowledge on several dimensions (e.g., fluency and flexibility), while schematic knowledge shows a specific advantage in originality. Conceptually, schematic knowledge emphasizes abstract principles and constraints (similar to formulas, theorems, and scientific definitions), which may support abstraction and flexible application beyond surface contexts. In contrast, case-based knowledge includes additional situational details and procedural steps. Although such details can aid comprehension in some learning tasks, they may also anchor learners’ representations to specific contexts or exemplars, thereby limiting the breadth of application during divergent ideation (de Chantal et al., 2025; Yuan et al., 2021). This interpretation is consistent with evidence highlighting how focusing on abstract conceptual features rather than concrete exemplars can promote more novel generation (Hao & Wu, 2010; Ward et al., 2002) and with work suggesting that strong encoding of exemplar details does not necessarily benefit creative idea generation (Z. Zhang et al., 2025). Thus, the efficacy of case-based knowledge largely depends on how it is implemented in teaching. For instance, when learners are encouraged to engage in inductive reasoning—that is, generating explanations of explanations or abstract principles from specific cases—case-based approaches may also foster creative thinking. Conversely, an instructional approach that emphasizes memorization or reproduction of case details may constrain idea generation. What’s more, we note that case-based approaches can be valuable in domains where theoretical knowledge is limited and experience-driven reasoning is critical (W. Liu et al., 2020; Althuizen & Wierenga, 2014; Guo et al., 2013). The relative effectiveness of knowledge forms also depends on whether the creative task demands principle-based transfer or context-specific problem solving.

Crucially, our knowledge-structure results provide a plausible mechanism for why schematic knowledge better supports scientific divergent thinking. The schematic condition tended to yield knowledge structures with higher clustering and efficiency and shorter paths, and these structural properties were consistently associated with SDAT performance. Even without a definitive indirect-effect test, the alignment among (i) knowledge-form differences in SDAT outcomes, (ii) knowledge-form differences in knowledge-structure metrics, and (iii) structure–outcome associations provides triangulated evidence consistent with the view that instructional form can shape scientific creativity partly by shaping learners’ internal knowledge organization. This helps bridge descriptive findings (“schematic works better”) with a mechanistic explanation that is actionable for educational design.

From an educational perspective, these findings suggest that fostering scientific creativity may require more than increasing knowledge quantity; it may require supporting learners to construct representations that are principle-centered, efficiently connected, and easier to traverse when generating applications (Benedek et al., 2023). In practice, this points to instructional strategies that emphasize schematic summaries of core mechanisms, explicit representation of constraints, and structured mapping of relations among principles—especially when the goal is to promote creative transfer to new applications.

### 4.3. Instructional Implications

In traditional knowledge-based learning processes that emphasize memorization, different forms of knowledge play varying roles in fostering scientific divergent thinking. From an instructional-design perspective, our findings suggest a goal-aligned approach to fostering scientific divergent thinking. For improving overall ideational productivity (overall score, fluency, flexibility), instructors may avoid relying primarily on case-based narratives and instead begin with representations that foreground relations among concepts (e.g., brief principle summaries, relational mapping prompts, structured concept-linking activities). In other words, presenting schematic knowledge and associative knowledge is likely to be a better choice in such situations than case-based knowledge. In practice, this may involve presenting key principles in a more structured and explicit manner (e.g., highlighting conceptual relations or constraints), rather than relying primarily on narrative cases.

When the goal is specifically to enhance originality, the results further suggest prioritizing schematic, principle-centered formats that make the causal mechanism and boundary conditions explicit (e.g., mechanism diagrams, constraint tables, “if–then” rules), which may help learners move beyond surface contexts and recombine principles with more remote domains. Cases can then be introduced as follow-up practice to test and refine the schema, rather than as the primary representation, thereby balancing plausibility with novelty.

### 4.4. Limitations and Directions for Future Research

Several limitations should be acknowledged in this study. First, ecological validity and transfer. Experiment 2 assessed scientific divergent thinking immediately after learning and did not include longitudinal assessments (e.g., delayed retention) or near/far transfer measures. Incorporating delayed tests and cross-topic transfer would clarify whether instructional knowledge form supports durable and transferable scientific creativity rather than short-lived performance gains. Furthermore, this study focuses solely on the form in which knowledge is presented and does not address other key instructional variables, such as the timing of knowledge presentation, the quality of example, or specific teacher guidance strategies. Future studies should test whether the observed advantages of schematic (and associative) formats persist over time and generalize to more authentic STEM outcomes, such as project-based design tasks, explanation-to-application assessments, or classroom assignments.

Second, task and measurement scope. Although the SDAT is designed to capture scientifically constrained divergent application, it represents only one operationalization of scientific creativity. Future work should triangulate across multiple tasks (e.g., scientific problem finding, hypothesis generation, design optimization) and examine whether knowledge-structure indices predict different creativity facets consistently (e.g., Beaty et al., 2026). In addition, extending scoring beyond fluency/flexibility/originality to include feasibility/appropriateness may better reflect the “novel-and-useful” criterion central to creativity in scientific contexts.

Third, mechanism and process evidence. Our results provide convergent evidence consistent with a knowledge-organization account, but a single-step mediation test did not yield a statistically reliable indirect effect. This suggests that the mechanism may be distributed across multiple correlated structural properties and/or influenced by unmeasured process variables (e.g., abstraction, strategy use, cognitive load). Future research should combine semantic memory network measures with process-sensitive methods (e.g., think-aloud protocols, strategy reports, time-on-task, eye tracking) and use model-based approaches (e.g., latent variable modeling or preregistered composite structure factors) to more directly test mechanistic pathways (Ganor et al., 2025).

In addition, although the core conceptual content across the three knowledge-form conditions was designed to be comparable, the materials differed somewhat in the amount of detail and informational complexity. More detailed descriptions may reduce participants’ tendency to elaborate or generate additional information on their own, which may have partially contributed to the observed differences in scientific divergent thinking performance. Future research should therefore more strictly match textual complexity and informational density across conditions. Moreover, semantic similarities between participant-generated concepts were estimated using a Word2Vec model trained on large-scale Chinese corpora. Although this approach enables comparable estimation of semantic relationships across participants, the resulting semantic space primarily reflects statistical regularities in general language use and may not fully capture highly contextualized or dynamic semantic associations. Future research may therefore benefit from incorporating newer semantic modeling approaches, such as contextualized embeddings or large language models.

Fourth, the present sample size was modest and consisted of relatively homogeneous Chinese undergraduate students, and the scientific materials were derived from contemporary inventions. Such sample characteristics may limit the generalizability of the findings, as reduced variability in background knowledge and educational experience may constrain the extent to which the observed effects generalize to more diverse populations. Generalization to different age groups (secondary students), expertise levels (novices vs. domain majors), and instructional contexts remains to be tested. The relative benefits of schematic versus case-based knowledge may vary depending on the instructional goal (principle-based transfer vs. context-specific problem solving), task constraints, and learners’ prior knowledge. Future studies should explicitly examine these moderators to identify when schematic instruction is most beneficial and when cases may be advantageous.

## 5. Conclusions

In sum, this study demonstrates that the organization of learners’ knowledge, quantified through semantic memory network metrics, is systematically related to scientific divergent thinking. By constructing semantic networks based on the semantic distances between participant-generated concepts and analyzing their graph-theoretical properties, the present study provides a quantitative approach to capturing how individuals organize knowledge. Moreover, when core content is held constant, schematic knowledge presentation enhances scientific creativity, particularly originality, and is accompanied by knowledge structures that are more clustered and efficient. Together, these findings highlight knowledge organization as a meaningful cognitive mechanism and suggest that schematic, principle-centered instruction may be a promising direction for fostering scientific creativity in STEM learning contexts.

## Figures and Tables

**Table 1:** Correlation between the scores on the SDAT and the AUT across dimensions.

	Overall—AUT	Fluency—AUT	Flexibility—AUT	Originality—AUT
Overall—SDAT	0.560 ***	0.583 ***	0.470 **	0.445 **
Fluency—SDAT	0.552 ***	0.601 ***	0.445 **	0.472 **
Flexibility—SDAT	0.538 ***	0.542 ***	0.454 **	0.391 *
Originality—SDAT	0.524 **	0.562 ***	0.406 *	0.458 **

Note: AUT stands for the Alternative Use Task, while SDAT refers to the Scientific Divergent Application Task. *—*p* < .05, **—*p* < .01, ***—*p* < .001.

**Table 2:** Correlation Between SDAT and Knowledge Structure Across Dimensions in Pilot Experiment.

	*M* ± *SD*	Overall—SDAT	Fluency—SDAT	Flexibility—SDAT	Originality—SDAT
Clustering coefficient	0.754 ± 0.030	0.317 *	0.312	0.331 *	0.284
Global efficiency	0.763 ± 0.028	0.316	0.329 *	0.332 *	0.304
Local efficiency	0.755 ± 0.029	0.317 *	0.317 *	0.331 *	0.289
Average shortest path length	1.353 ± 0.057	–0.314	–0.317 *	–0.326 *	–0.285

Note: SDAT stands for the Scientific Divergent Application Task. *—*p* < .05.

**Table 3:** Correlation Between SDAT and Knowledge Structure Across Dimensions in Experiment 2.

	*M* ± *SD*	Overall—SDAT	Fluency—SDAT	Flexibility—SDAT	Originality—SDAT
Clustering coefficient	0.762 ± 0.021	0.214 *	0.198 *	0.209 *	0.279 **
Global efficiency	0.770 ± 0.020	0.222 *	0.208 *	0.216 *	0.280 **
Local efficiency	0.763 ± 0.020	0.215 *	0.200 *	0.211 *	0.280 **
Average shortest path length	1.338 ± 0.040	–0.210 *	–0.199 *	–0.207 *	–0.279 **

Note: SDAT stands for the Scientific Divergent Application Task. Values in parentheses are *p* values. *—*p* < .05, **—*p* < .01.

**Table 4:** Hierarchical regression of knowledge structure on SDAT scores.

Predictors	Overall β	Fluency β	Flexibility β	Originality β
Model 1				
Associative (vs. schematic)	−0.089	0.038	0.011	−0.369 ***
Case-based (vs. schematic)	−0.461 ***	−0.289 **	−0.333 ***	−0.446 ***
*R* ^2^	0.180 ***	0.096 **	0.115 ***	0.171 ***
Model 2				
Associative (vs. schematic)	−0.039	0.090	0.065	−0.315 **
Case-based (vs. schematic)	−0.432 ***	−0.258 **	−0.301 **	−0.415 ***
Knowledge structure	0.199 *	0.211 *	0.215 *	0.215 **
*R* ^2^	0.217 ***	0.138 ***	0.159 ***	0.215 ***
∆*R*^2^	0.038 *	0.042 *	0.044 *	0.044 **

Note: SDAT stands for the Scientific Divergent Application Task. Standardized regression coefficients (β) are reported. Knowledge form was dummy-coded with schematic knowledge as the reference group. Knowledge structure represents a composite index computed by standardizing and averaging clustering coefficient, global efficiency, local efficiency, and reversed average shortest path length. Δ*R*^2^ indicates the increase in explained variance after adding knowledge-structure indices in Model 2. *—*p* < .05, **—*p* < .01, ***—*p* < .001.

## Data Availability

The data presented in this study are available on request from the corresponding author. The data are not publicly available due to privacy and ethical restrictions related to participant information.

## Supplementary Materials

The following supporting information can be downloaded at: https://www.mdpi.com/article/10.3390/jintelligence14070118/s1, Supplementary File S1: Learning materials in the SDAT.
