# Supplementary material for: How Knowledge Structure and Form Shape Scientific Divergent Thinking: Evidence from Semantic Network Analysis and the Scientific Divergent Application Task
Source: J Intell. 2026 Jun 25;14(7):118. doi: 10.3390/jintelligence14070118 (PMC13413292; doi:10.3390/jintelligence14070118)
Supplement: Supplementary file 1 [file jintelligence-14-00118-s001.zip › jintelligence-4259320-supplementary.pdf]

S1: Learning materials in the SDAT .

Learning materials in the SDAT

1. **A alkene**: a nearly transparent novel material that is reportedly among the lightest and hardest substances known, with excellent electrical and thermal conductivity.

Application Scenarios:

- Supercomputer components currently lack sufficient conductivity, with electron mobility remaining low at room temperature.
- Sensor surface coatings must be transparent and lightweight yet resistant to damage.
- Thinner smartphone screens are prone to breakage, while thicker ones sacrifice transparency and suffer from reduced responsiveness due to poor thermal conductivity.
- Body armor remains heavy and bulky, with suboptimal toughness and strength.

2. **C beam** offers advantages such as high destructive power, resistance to electromagnetic interference, controllable beam diameter, and high beam intensity.

Application Scenarios:

- The lithography process requires drilling very small holes in components, which is difficult to achieve using conventional techniques in the manufacture of integrated circuits.
- Traditional chemical etching techniques are complex and cannot be used to process certain specialized materials.
- Communications are prone to electromagnetic interference in environments with strong interference.
- When measuring distance, it is necessary to accurately measure the distance between two points; generally, light has poor convergence.

3. **H oxygen** offers antibacterial properties, odor elimination, the ability to adsorb impurities and toxins from water, and portability.

Application Scenarios:

- In emergency medical care, oxygen supply equipment is often bulky and difficult to move.

- The enclosed environment inside a car, with its low oxygen levels and unpleasant odors, can pose a health risk.
- Air purifiers need to sterilize the air to prevent bacterial infections.
- Fish often die in aquariums due to oxygen deprivation and the unchecked growth of harmful bacteria.

4. **Y mud** is characterized by its wear resistance, non-toxicity, smooth surface, and noise-reducing properties.

Application Scenarios:

- Hospitals require a quiet, sterile environment to facilitate patient recovery; relying solely on patients' self-discipline and reminders has proven ineffective.
- There needs to be good soundproofing between the screening rooms in a movie theater.
- Rails frequently carry sharp-edged objects, so high wear resistance is required to minimize rail wear.
- When installing flooring, uneven subfloors often lead to excessive wear and tear.

5. **P glue** is mild and non-irritating, with a certain degree of elasticity and softness, and exhibits adsorption properties toward small molecules.

Application Scenarios:

- Babies are very sensitive to external stimuli, so pacifiers must be made of non-irritating materials.
- Beer often contains tiny protein particles that cause it to become cloudy and affect its color.
- Cosmetic surgery requires the insertion of materials into the body that are similar to human tissue and possess elasticity and softness.
- A vacuum extractor can help to prevent intracranial injury to the foetus during delivery. The material used must be sufficiently elastic, soft and non-irritating.
